# A nonlinear relationship between alcohol consumption and cognitive function in elderly people: a population-based study from NHANES 2011–2014

**DOI:** 10.3389/fnagi.2024.1458274

**Published:** 2024-11-25

**Authors:** Kaiqi Chen, Yunhua Li, Rui Yue, Zhao Jin, Shikui Cui, Xijian Zhang, Danping Zhu, Qihui Li

**Affiliations:** ^1^School of Basic Medical, Chengdu University of Traditional Chinese Medicine, Chengdu, China; ^2^College of Education, Chengdu College of Arts and Sciences, Chengdu, China; ^3^Department of Traditional Chinese Medicine, Chongqing Changhang Hospital, Chongqing, China; ^4^Department of Endocrinology, Chongqing Traditional Chinese Medicine Hospital, Chongqing, China; ^5^Department of Nephrology, Chongqing Traditional Chinese Medicine Hospital, Chongqing, China

**Keywords:** NHANES, alcohol intake, cognitive function, association, cross-sectional analysis

## Abstract

**Objective:**

This study aims to explore the association between alcohol intake and cognitive function in elderly Americans, including potential nonlinear relationships and interactions across different subgroups.

**Methods:**

The study analyzed data from the National Health and Nutrition Examination Survey (NHANES) from 2011 to 2014. The sample included 2,675 Americans aged 60 or older. Multivariate regression analysis was used to evaluate the relationship between alcohol intake and cognitive function. Smooth curve fitting and threshold effect analysis were employed to explore potential nonlinear relationships. Subgroup analyses were conducted to examine the stability of the results across different subgroups.

**Results:**

The results indicate a significant negative correlation between alcohol intake and cognitive function. In the CERAD total word recall test, for every unit increase in alcohol intake, the score decreased by 0.15 points (−0.15, 95% CI: −0.25, −0.04), and in the CERAD delayed recall test, it decreased by 0.07 points (−0.07, 95% CI: −0.12, −0.01). Compared to Non-Heavy Drinkers, Heavy Drinkers showed a reduction in their CERAD total word recall scores by-0.77 points (−0.77, 95% CI: −1.23, −0.32) and in their CERAD delayed recall scores by-0.28 points (−0.28, 95% CI: −0.52, −0.04). Smooth curve fitting analysis revealed a nonlinear relationship between alcohol intake and cognitive function, with breakpoints at 10.7 for the CERAD total word recall test, 4.7 for the Animal fluency test, and 3.85 for the Digit symbol substitution test. Additionally, subgroup analysis indicated that gender, educational level, and smoking status significantly moderated the relationship between alcohol intake and cognitive function, while marital status, race, hypertension, diabetes, and cancer status showed no significant interactions.

**Conclusion:**

The association between alcohol intake and cognitive function in the elderly is complex, influenced by both the amount of intake and individual subgroup characteristics.

## 1 Introduction

With the aging of the global population, the prevalence and negative impacts of cognitive impairment among the elderly have become increasingly evident (Wang et al., 2020). Cognitive impairment not only compromises the quality of life of the elderly but also imposes significant economic burdens on society and families (Seijo-Martinez et al., 2016; Tahami Monfared et al., 2022; Hill et al., 2017). Among the many factors influencing cognitive health in the elderly, alcohol intake is a significant yet controversial issue (Jiang et al., 2023; Wang et al., 2020).

Alcohol intake refers to the consumption of alcoholic beverages (Zhao et al., 2023), a practice that is widespread globally. According to the World Health Organization (2018), approximately two billion people worldwide consume alcohol. Large-scale surveys in high-income countries indicate an increasing trend of alcohol consumption among the elderly, encompassing moderate, frequent, and problematic excessive drinking patterns (Han et al., 2019; Huhn et al., 2019). While moderate alcohol consumption may have beneficial effects on cardiovascular health, excessive intake has been consistently linked to a range of health problems, notably cognitive decline, in the elderly population (Zhang et al., 2020; Yen et al., 2022; Mezue et al., 2023). In the elderly, alcohol is metabolized more slowly, rendering even moderate intake potentially harmful to cognitive function due to its direct toxic effects on brain structures, particularly the hippocampus and frontal lobes (Kmietowicz, 2017; Topiwala and Ebmeier, 2018).

Cognitive function, a critical factor for the health and independent living of the elderly, encompasses multiple dimensions such as memory, attention, and executive functions (Portaccio, 2018). According to the Alzheimer’s Association in the United States, approximately 50 million people worldwide are affected by various forms of cognitive impairments, and this number is expected to double in the coming decades (Alzheimer's Association, 2019). Beyond memory loss, cognitive decline also encompasses reduced decision-making abilities, impaired problem-solving skills, and language deficits, all significantly affecting the daily lives of the elderly (Clare et al., 2017; Sun et al., 2023; Cohen et al., 2023).

Although existing studies provide some insights into the relationship between alcohol intake and cognitive function, the results are often contradictory. Some studies suggest that low to moderate alcohol intake may have a protective effect on cognition (Zhang et al., 2020), while other studies indicate that alcohol consumption is associated with accelerated cognitive decline (Topiwala et al., 2017). Therefore, this study aims to explore the relationship between alcohol intake and cognitive function among the elderly using data from the National Health and Nutrition Examination Survey (NHANES) database for the years 2011–2014. We hope to provide a more precise and reliable assessment of this relationship through multivariate adjustments and a large sample size, aiming to fill the gaps in the existing literature.

## 2 Materials and methods

### 2.1 Study population and design

**Figure 1:**
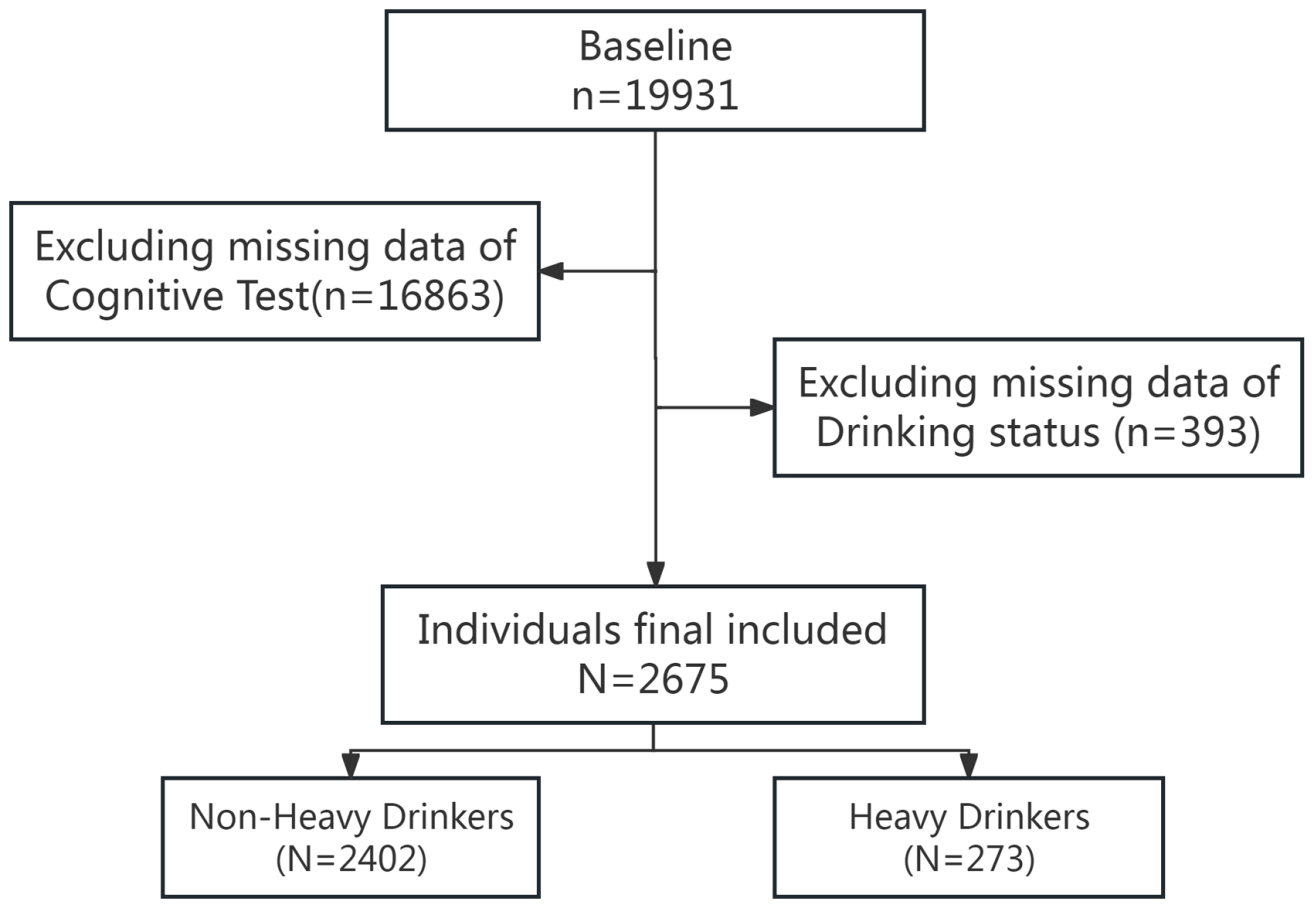
Flow chart of the screening process for the selection of eligible participants in NHANES 2011–2014.

This study utilized data from the NHANES survey conducted by the National Center for Health Statistics (NCHS). The NHANES survey received ethical approval from the NCHS Ethics Review Committee, and all participants provided informed consent. This study analyzed data from two consecutive two-year cycles of the NHANES survey (2011–2014). The inclusion criteria for participants in this study were as follows: (Wang et al., 2020) participants aged 60 years or older; (Seijo-Martinez et al., 2016) individuals missing cognitive function scores were excluded; (Tahami Monfared et al., 2022) participants missing alcohol intake data were also excluded. Initially, 19,931 individuals were recruited. We then excluded individuals missing cognitive test data, retaining a cohort of 3,068 elderly Americans. Subsequently, individuals with incomplete drinking information were excluded, resulting in a final sample of 2,675 participants (Figure 1).

### 2.2 Alcohol assessment

In our study, the assessment of alcohol consumption utilized the Automated Multiple-Pass Method (AMPM) (Blanton et al., 2006), a detailed dietary recall technique from the NHANES. This method enhances the accuracy of dietary data collection, including a specific protocol to capture all consumed foods and beverages, with a focus on the quantity and context of consumption (Blanton et al., 2006). The AMPM has been proven effective in collecting energy intake data in adult populations in previous large studies (Raper et al., 2004; Moshfegh et al., 2008).

Dietary recall involves two sequential parts: an initial in-person interview followed by a telephone interview 3–10 days later (Waxman and World Health Assembly, 2004). Both sessions employ the AMPM to comprehensively capture typical food and beverage intake over a 24-h period (from midnight to midnight) (Blanton et al., 2006). During the first interview conducted at NHANES Mobile Examination Centers (MEC), participants use various physical measuring guides to accurately report the intake of food and beverages, including alcoholic drinks. These guides include replicas of standard glasses, cups, and bottles to help quantify liquid intake, as detailed in the “NHANES Measuring Guides for the Dietary Recall Interview” (Centers for Disease Control and Prevention, 2006). Additionally, to specifically assess alcohol consumption, the interview queries the type and context of the drinking beverages to ensure detailed and accurate data collection. For coding and analysis, NHANES employs the USDA’s Food and Nutrient Database for Dietary Studies (FNDDS), which includes comprehensive nutrient data and facilitates the calculation of alcohol intake in grams (Ahuja et al., 2013). This system uses the latest nutrient values reflecting current food supply and consumption patterns (Matthews et al., 2016). By adopting these rigorous methods, the study ensures that the data on alcohol intake is both accurate and reflective of the participants’ usual consumption.

According to the U.S. Dietary Guidelines for Alcohol (USDGA), it is recommended that individuals limit their daily alcohol intake to no more than 14 grams for women (1 standard alcoholic drink per day) and 28 grams for men (2 standard alcoholic drinks per day) (Matthews et al., 2016). Individuals exceeding these guidelines were defined as heavy drinkers.

### 2.3 Measurement of cognitive function

The Consortium to Establish a Registry for Alzheimer’s Disease (CERAD W-L) assesses an individual’s ability to acquire and retain new verbal material through immediate and delayed recall tasks (Fillenbaum and Mohs, 2023; Paajanen et al., 2014). The CERAD W-L protocol includes three consecutive learning trials and one delayed recall task. During the three learning trials, participants are instructed to verbally express a list of 10 unrelated words. After the words are presented, participants immediately recall as many words as possible. The delayed recall occurs approximately 10 min after the start of the word learning trials. Each test has a maximum score of 10 points, with the total score for the three tests and delayed recall ranging from 0 to 40 points. The Animal Fluency Test (AFT) assesses the fluency of language categories, which is a fundamental aspect of executive function (Carone et al., 2007). Additionally, the test also evaluates other capabilities related to semantic memory and processing speed. Participants score a point for each animal they name, and they are asked to list as many animals as possible within 1 min. The Digit Symbol Substitution Test (DSST) is a comprehensive assessment tool for evaluating brain health, covering multiple cognitive domains such as visual scanning, processing speed, short-term memory, and sustained attention (Proust-Lima et al., 2007; Bienias et al., 2003). The test utilizes a paper form with a key at the top that features nine numbers paired with different symbols. Participants have 2 min to replicate characters associated with 133 boxes next to numbers. Their scores range from 0 to 133, reflecting the cumulative number of correct matches. Higher scores indicate stronger cognitive abilities across all assessments. Currently, there are no universally accepted standards for defining cognitive impairment levels using the CERAD, AFT, and DSST tests. Therefore, related studies typically use the lowest quartile of scores for each test as the threshold for detecting cognitive impairment, a practice consistent with previous research literature (Kou et al., 2023; Dong et al., 2020; Zhang et al., 2024).

### 2.4 Covariables

The covariates considered in the study include age (in years), gender (categorized as male or female), race (including Mexican American, other Hispanic, non-Hispanic white, non-Hispanic black, and other races), marital status classified into married/living with partner, widowed/divorced/separated, and never married, education level (less than high school, high school, and more than high school), income relative to the poverty index (PIR), body mass index (BMI), smoking status (indicated by yes or no for smoking more than 100 cigarettes in lifetime), diabetes status (indicated by yes or no), hypertension status (indicated by yes or no), congestive heart failure (indicated by yes or no), coronary heart disease (indicated by yes or no), angina (indicated by yes or no), heart attack (indicated by yes or no), stroke status (indicated by yes or no), and cancer (indicated by yes or no). The NHANES survey methods and analytic guidelines provide comprehensive data on various methods for collecting variables.^1^

### 2.5 Statistical analysis

All analyses were performed using R software version 4.2 and EmpowerStats version 4.1. Based on alcohol consumption, we categorized the sample into two groups: heavy drinkers and non-heavy drinkers. This classification enabled us to evaluate the demographic differences between the groups using rank-sum tests and t-tests, aiming to explore the potential effects of varying drinking intensities on cognitive function. Continuous variables were represented as means ± standard deviations (SD), and categorical variables were presented as counts and percentages (n (%)). To investigate the linear relationship between alcohol intake and cognitive function, we utilized weighted multivariate linear regression models. Specifically, we developed three multivariate models for testing: Model 1 made no adjustments; Model 2 adjusted for factors such as gender, age, race, educational level, and marital status; Model 3 further adjusted for all covariates. These models allowed us to more precisely evaluate the relationship between alcohol intake and cognitive function, taking into account potential confounders.

## 3 Results

### 3.1 Baseline characteristics

Demographic characteristics stratified by alcohol intake quintiles are presented in Table 1. The study included 2,675 participants aged 60 or older, comprising 1,310 males (49%) and 1,365 females (51%). The average age (SD) and average alcohol intake (SD) of the 2,775 participants were 69.4 (6.8) years and 5.66 (1.53) grams per day, respectively. The alcohol intake quintiles for females and males were ≤ 14 grams/day (1 standard alcoholic drink/day) and ≤ 28 grams/day (2 standard alcoholic drinks/day), respectively. Compared to the Non-Heavy Drinkers group, the Heavy Drinkers group had higher odds of being younger, having lower BMI, belonging to three ethnic groups, being unmarried, having three education levels, and smoking more cigarettes (Table 1).

**Table 1:** Baseline characteristics of the study population according to alcohol intake.

	Overall	Non-Heavy drinkers	Heavy drinkers	*p*-value
N	2,675	2,402	273	
Age (Years)	69.4 ± 6.8	69.5 ± 6.8	68.5 ± 6.6	0.020
PIR^1^	2.6 ± 1.5	2.6 ± 1.5	3.1 ± 1.6	<0.001
BMI^2^	29.1 ± 6.3	29.3 ± 6.4	27.5 ± 5.8	<0.001
Alcohol intake (gm)	5.66 ± 1.54	2.0 ± 0.5	41.0 ± 2.7	<0.001
CERAD^3^ total word recall	19.0 ± 4.6	18.9 ± 4.6	19.3 ± 4.4	0.217
CERAD delayed recall	5.9 ± 2.3	5.9 ± 2.3	6.0 ± 2.2	0.583
Animal fluency test	16.6 ± 5.4	16.5 ± 5.5	17.7 ± 5.3	<0.001
Digit symbol substitution test	46.1 ± 17.1	45.5 ± 17.1	51.3 ± 15.9	<0.001
Gender				0.498
Male	1,310 (49.0%)	1,171 (48.8%)	139 (50.9%)	
Female	1,365 (51.0%)	1,231 (51.2%)	134 (49.1%)	
Race				<0.001
Mexican American	232 (8.7%)	211 (8.8%)	21 (7.7%)	
Other Hispanic	272 (10.2%)	252 (10.5%)	20 (7.3%)	
Non-Hispanic White	1,310 (49.0%)	1,135 (47.3%)	175 (64.1%)	
Non-Hispanic Black	632 (23.6%)	580 (24.1%)	52 (19.0%)	
Other races	229 (8.6%)	224 (9.3%)	5 (1.8%)	
Marital status				0.659
Married/living with partner	1,557 (58.2%)	1,392 (58.0%)	165 (60.4%)	
Widowed/divorced/separated	967 (36.1%)	872 (36.3%)	95 (34.8%)	
Never married	151 (5.6%)	138 (5.7%)	13 (4.8%)	
Education Level				<0.001
Less than high school	664 (24.8%)	623 (25.9%)	41 (15.0%)	
High school or GED	630 (23.6%)	574 (23.9%)	56 (20.5%)	
Above high school	1,381 (51.6%)	1,205 (50.2%)	176 (64.5%)	
Had at least 100 cigarettes/life				<0.001
No	1,307 (48.9%)	1,213 (50.5%)	94 (34.4%)	
Yes	1,368 (51.1%)	1,189 (49.5%)	179 (65.6%)	
High blood pressure				0.025
No	1,000 (37.4%)	881 (36.7%)	119 (43.6%)	
Yes	1,675 (62.6%)	1,521 (63.3%)	154 (56.4%)	
Diabetes				<0.001
No	1922 (71.9%)	1,684 (70.1%)	238 (87.2%)	
Yes	631 (23.6%)	606 (25.2%)	25 (9.2%)	
Borderline	122 (4.6%)	112 (4.7%)	10 (3.7%)	
Congestive heart failure				0.107
No	2,484 (92.9%)	2,224 (92.6%)	260 (95.2%)	
Yes	191 (7.1%)	178 (7.4%)	13 (4.8%)	
Coronary heart disease				0.092
No	2,423 (90.6%)	2,168 (90.3%)	255 (93.4%)	
Yes	252 (9.4%)	234 (9.7%)	18 (6.6%)	
Angina				0.758
No	2,527 (94.5%)	2,268 (94.4%)	259 (94.9%)	
Yes	148 (5.5%)	134 (5.6%)	14 (5.1%)	
Heart attack				0.106
No	2,438 (91.1%)	2,182 (90.8%)	256 (93.8%)	
Yes	237 (8.9%)	220 (9.2%)	17 (6.2%)	
Stroke				0.139
No	2,490 (93.1%)	2,230 (92.8%)	260 (95.2%)	
Yes	185 (6.9%)	172 (7.2%)	13 (4.8%)	
Cancer				0.814
No	2,131 (79.7%)	1915 (79.7%)	216 (79.1%)	
Yes	544 (20.3%)	487 (20.3%)	57 (20.9%)	

Mean ± SD for continuous variables: the *p* value was calculated by the weighted linear regression model; (%) for categorical variables: the *P* value was calculated by the weighted chi-square test.

1 PIR, the ratio of income to poverty.

2 BMI, Body Mass Index.

3 CERAD, Consortium to Establish a Registry for Alzheimer’s Disease.

### 3.2 Association between alcohol intake and cognitive function

Our study findings indicate that higher alcohol intake is associated with an increased risk of cognitive impairment. In the multivariate regression analysis, with alcohol intake treated as a continuous variable and progressively adjusted, this association was significant in Model 3 with full covariate adjustment for the CERAD total word recall (OR = −0.15, 95% CI: −0.25, −0.04) and for the CERAD delayed recall (OR = −0.07, 95% CI: −0.12, −0.01). This suggests that for each unit increase in alcohol intake, the cognitive function scores decrease by-0.15 and-0.07 points in the fully adjusted models for these two tests, respectively. However, this relationship was not significant in AFT and DSST. When categorizing alcohol intake into Non-Heavy Drinkers and Heavy Drinkers, in the CERAD total word recall, the latter group had a 77% greater reduction in cognitive function scores compared to the former (OR = −0.77, 95% CI: −1.23, −0.32), and in the CERAD delayed recall, the latter group had a 28% greater reduction (OR = −0.28, 95% CI: −0.52, −0.04) (see Table 2).

**Table 2:** Association between alcohol intake and cognitive function.

Variables	Model 1	*P* value	Model 2	*P* value	Model 3	*P* value
CERAD^1^ total word recall	−0.05 (−0.16, 0.06)	0.402	−0.12 (−0.23, −0.02)	0.018	−0.15 (−0.25, −0.04)	0.006
Non-heavy drinkers	ref		ref		ref	
Heavy drinkers	0.08 (−0.41, 0.57)	0.743	−0.57 (−1.02, −0.13)	0.012	−0.77 (−1.23, −0.32)	<0.001
CERAD delayed recall	−0.03 (−0.09, 0.03)	0.302	−0.06 (−0.11, −0.00)	0.037	−0.07 (−0.12, −0.01)	0.016
Non-heavy drinkers	ref		ref		ref	
Heavy drinkers	0.09 (−0.17, 0.34)	0.505	−0.19 (−0.43, 0.04)	0.101	−0.28 (−0.52, −0.04)	0.021
Animal fluency test	0.28 (0.14, 0.42)	<0.001	0.01 (−0.12, 0.14)	0.895	−0.01 (−0.14, 0.12)	0.862
Non-heavy drinkers	ref		ref		ref	
Heavy drinkers	0.97 (0.35, 1.59)	0.002	−0.16 (−0.71, 0.40)	0.582	−0.40 (−0.97, 0.17)	0.166
Digit symbol substitution test	0.82 (0.40, 1.24)	<0.001	0.09 (−0.23, 0.41)	0.576	−0.02 (−0.35, 0.30)	0.889
Non-heavy drinkers	ref		ref		ref	
Heavy drinkers	5.19 (3.37, 7.01)	<0.001	1.16 (−0.26, 2.58)	0.108	0.02 (−1.38, 1.42)	0.980

Model 1: no covariates were adjusted. Model 2: Age; Gender; Race; Martial; Education. Model 3: Age; Gender; Race; Martial; Education; the ratio of income to poverty; Body mass index; smoking status; Blood pressure; Diabetes; Congestive heart failure; Coronary heart disease; Angina; Heart Attack; Stroke; Cancer were adjusted.

1 CERAD, Consortium to Establish a Registry for Alzheimer’s Disease.

**Figure 2:**
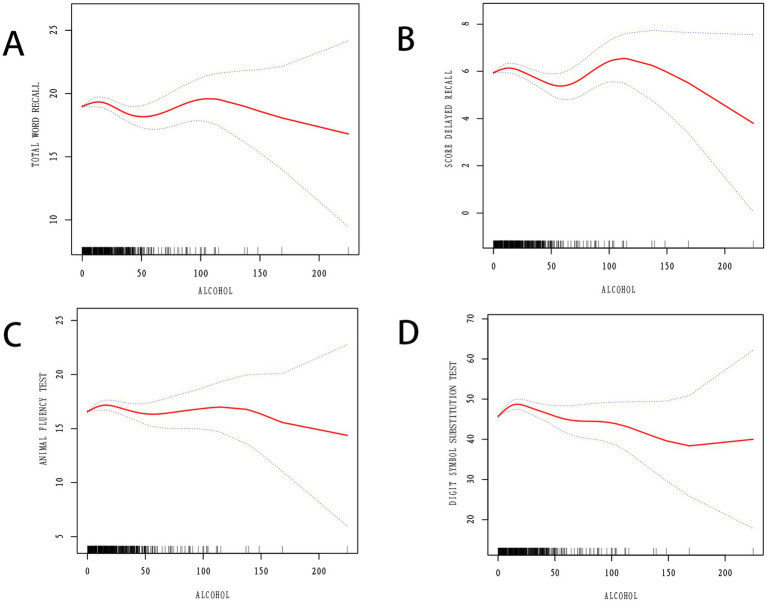
**(A)** Relationship between alcohol intake and CERAD total word recall by smooth curve fitting. **(B)** Relationship between alcohol intake and CERAD delayed recall by smooth curve fitting. **(C)** Relationship between alcohol intake and Animal fluency test by smooth curve fitting. **(D)** Relationship between alcohol intake and Digit symbol substitution test by smooth curve fitting.

Furthermore, this study utilized smooth curve fitting and threshold effect analysis to detect potential nonlinear relationships between alcohol intake and cognitive function. The results indicate a clear nonlinear curve relationship between alcohol intake and cognitive function (Figure 2). In the CERAD total word recall, the threshold point for alcohol intake, as determined by threshold effect analysis, was 10.7. The two-part linear regression model shows that the relationship between alcohol intake and cognitive function is not significant when alcohol intake is less than or equal to 10.7; however, for every unit increase in alcohol intake above 10.7, cognitive function decreases by 0.03 points (OR = −0.03, 95% CI: −0.04, −0.01, *p* = 0.001). The segmented logistic regression model outperforms the linear logistic regression model (*p* = 0.045) (Table 3). In the Animal Fluency Test, the threshold point for alcohol intake determined by threshold effect analysis was 4.7. The two-part linear regression model shows that for every unit increase in alcohol intake up to 4.7, cognitive function increases by 0.22 points (OR = 0.22, 95% CI: 0.11, 0.34, *p* < 0.001); however, for every unit increase in alcohol intake above 4.7, cognitive function decreases by 0.03 points (OR = −0.03, 95% CI: −0.04, −0.01, *p* = 0.006). The segmented logistic regression model outperforms the linear logistic regression model (*p* < 0.001) (Table 3). In DSST, the threshold point for alcohol intake determined by threshold effect analysis was 3.85. The two-part linear regression model shows that for every unit increase in alcohol intake up to 3.85, cognitive function increases by 0.97 points (OR = 0.97, 95% CI: 0.61, 1.33, *p* < 0.001); however, for every unit increase in alcohol intake above 3.85, cognitive function decreases by 0.09 points (OR = −0.09, 95% CI: −0.13, −0.04, *p* < 0.001). The segmented logistic regression model outperforms the linear logistic regression model (*p* < 0.001) (Table 3).

**Table 3:** Threshold analysis for the relationship between alcohol intake and cognitive function.

Models	CERAD^1^ total word recall	CERAD delayed recall	Animal fluency test	Digit symbol substitution test
Model I				
One line slope	−0.15 (−0.25, −0.04)	−0.07 (−0.12, −0.01)	−0.01 (−0.14, 0.12)	−0.02 (−0.35, 0.03)
Model II				
Turning point (K)	10.7	12.7	4.7	3.85
< K	0.03 (−0.02, 0.07) *p* = 0.2035	0.01 (−0.01, 0.03) *p* = 0.2582	0.22 (0.11, 0.34) *P* < 0.001	0.97 (0.61, 1.33) *P* < 0.001
> K	−0.03 (−0.04, −0.01) *P* = 0.001	−0.01 (−0.02, −0.00) *P* = 0.002	−0.03 (−0.04, −0.01) *P* = 0.006	−0.09 (−0.13, −0.04) *P* < 0.001
OR between <K and > K	−0.05 (−0.11, −0.00) *P* = 0.046	−0.02 (−0.05, 0.00) *p* = 0.0580	−0.25 (−0.38, −0.12) *P* < 0.001	−1.06 (−1.44, −0.67) *P* < 0.001
Logarithmic likelihood ratio test	0.045	0.057	<0.001	< 0.001

Adjust for: age; gender; race; martial; education; PIR; BMI; smoking status; blood pressure; diabetes; congestive heart failure; coronary heart disease; angina; heart attack; stroke; cancer.

1 CERAD, Consortium to Establish a Registry for Alzheimer’s Disease.

### 3.3 Subgroup analysis

Subgroup analysis using Model 3 further confirmed the stability of the relationship between alcohol intake and cognitive function across different populations (Figure 2). This study conducted subgroup and interaction analyses on the relationship between alcohol intake and cognitive function based on gender, marital status, race, education, smoking, hypertension, diabetes, and cancer. The results of the subgroup analysis showed no significant effects between alcohol intake and cognitive function across different strata in the interaction analyses for marital status, race, hypertension, diabetes, and cancer (P for interaction >0.05). However, significant interactions were observed in the interaction analyses for gender, education level, and smoking, indicating that the relationship between alcohol intake and cognitive function varied significantly across these strata (P for interaction <0.05) (see Table 4).

**Table 4:** Subgroup analysis of the association between alcohol intake with cognitive function.

Subgroup	CERAD^1^ total word recall β (95%CI)	P for interaction	CERAD delayed recall *β* (95%CI)	P for interaction	Animal fluency test β (95%CI)	P for interaction	Digit symbol substitution test β (95%CI)	P for interaction
Gender		0.751		0.540		0.389		0.022
Male	−0.14 (−0.26, −0.02)		−0.06 (−0.12, 0.00)		−0.04 (−0.19, 0.11)		−0.22 (−0.59, 0.15)	
Female	−0.18 (−0.39, 0.04)		−0.10 (−0.21, 0.01)		0.09 (−0.18, 0.35)		0.64 (−0.02, 1.29)	
Marital status		0.178		0.148		0.180		0.486
Married/living with partner	−0.21 (−0.34, −0.09)		−0.10 (−0.16, −0.03)		−0.08 (−0.24, 0.08)		−0.15 (−0.54, 0.24)	
Widowed/divorced/separated	−0.01 (−0.20, 0.18)		0.01 (−0.09, 0.11)		0.08 (−0.15, 0.32)		0.27 (−0.31, 0.86)	
Never married	0.00 (−0.49, 0.49)		−0.13 (−0.39, 0.12)		0.42 (−0.18, 1.03)		−0.03 (−1.53, 1.47)	
Ethnicity		0.605		0.478		0.478		0.604
Non-Hispanic White	0.14 (−0.59, 0.87)		0.12 (−0.26, 0.50)		0.12 (−0.26, 0.50)		0.48 (−1.75, 2.71)	
Non-Hispanic Black	0.10 (−0.46, 0.67)		0.08 (−0.21, 0.37)		0.08 (−0.21, 0.37)		0.14 (−1.58, 1.87)	
Mexican American	−0.18 (−0.29, −0.06)		−0.09 (−0.15, −0.03)		−0.09 (−0.15, −0.03)		−0.08 (−0.43, 0.28)	
Other Hispanic	−0.06 (−0.38, 0.26)		−0.01 (−0.18, 0.15)		−0.01 (−0.18, 0.15)		−0.03 (−1.02, 0.96)	
Other race	0.20 (−0.61, 1.02)		0.12 (−0.30, 0.54)		0.12 (−0.30, 0.54)		1.93 (−0.56, 4.42)	
Education level		0.02		0.046		0.122		0.628
Less than high school	0.19 (−0.12, 0.51)		0.07 (−0.09, 0.24)		0.26 (−0.13, 0.65)		0.07 (−0.89, 1.04)	
High school or GED	0.03 (−0.16, 0.22)		−0.01 (−0.11, 0.09)		−0.19 (−0.43, 0.05)		−0.27 (−0.86, 0.33)	
Above high school	−0.29 (−0.42, −0.15)		−0.12 (−0.19, −0.05)		0.02 (−0.14, 0.19)		0.07 (−0.34, 0.48)	
Had at least 100 cigarettes/life		0.153		0.391		0.027		0.044
No	−0.27 (−0.46, −0.07)		−0.05 (−0.12, 0.01)		−0.10 (−0.25, 0.05)		−0.21 (−0.59, 0.16)	
Yes	−0.10 (−0.22, 0.02)		−0.11 (−0.21, −0.01)		0.22 (−0.02, 0.46)		0.50 (−0.11, 1.10)	
High blood pressure		0.435		0.145		0.863		0.203
No	−0.10 (−0.26, 0.05)		−0.03 (−0.10, 0.05)		−0.02 (−0.21, 0.17)		−0.24 (−0.71, 0.23)	
Yes	−0.18 (−0.32, −0.04)		−0.10 (−0.18, −0.03)		−0.00 (−0.18, 0.17)		0.16 (−0.27, 0.59)	
Diabetes		0.993		0.429		0.174		0.486
No	−0.03 (−0.15, 0.08)		−0.03 (−0.09, 0.03)		−0.02 (−0.15, 0.12)		0.02 (−0.32, 0.36)	
Yes	−0.05 (−0.45, 0.35)		0.04 (−0.16, 0.24)		0.04 (−0.43, 0.51)		0.77 (−0.43, 1.96)	
Borderline	−0.06 (−0.43, 0.32)		0.08 (−0.10, 0.27)		0.41 (−0.02, 0.85)		−0.02 (−1.13, 1.09)	
Cancer		0.875		0.689		0.465		0.888
No	−0.03 (−0.15, 0.08)		−0.01 (−0.07, 0.04)		0.04 (−0.09, 0.17)		0.06 (−0.29, 0.40)	
Yes	−0.06 (−0.32, 0.20)		−0.04 (−0.17, 0.09)		−0.08 (−0.39, 0.22)		**2.21 (−0.66, 0.89)**	

1 CERAD, Consortium to Establish a Registry for Alzheimer’s Disease.

## 4 Discussion

This study, based on data from NHANES 2011–2014, thoroughly investigated the relationship between alcohol intake and cognitive function among elderly Americans, finding a significant negative correlation between alcohol intake and cognitive impairment. Through multivariate regression analysis, we confirmed that with every unit increase in alcohol intake, cognitive function scores significantly decreased by 0.15 and 0.07 points in the CERAD total word recall and delayed recall tests, respectively. Moreover, our research revealed a nonlinear relationship between alcohol intake and cognitive function, indicating that in some cognitive tests, such as the Animal Fluency Test and the Digit Symbol Substitution Test, the impact of alcohol intake on cognitive function switches from positive to negative once a certain threshold is reached. Through subgroup analysis, this study further confirmed the stability and variability of this relationship across different populations (such as different education levels and smoking status), providing insights into how alcohol intake can affect elderly cognitive health through various physiological and psychological mechanisms.

The multivariate regression analysis of this study reveals a significant negative correlation between alcohol intake and cognitive impairment in the elderly population, highlighting the potential risk of alcohol consumption on cognitive health. This finding is consistent with the meta-analysis by Spinola et al., which found a small to moderate negative impact of alcohol intake on working memory across 32 studies involving 1,629 participants (Anstey et al., 2019). Furthermore, research by Gong et al. (2021) demonstrated through maze experiments how higher doses of alcohol intake increase the production and deposition of beta-amyloid (Aβ) in the hippocampus and cerebral cortex of rats, clearly indicating that the accumulation of this biomarker is closely associated with the decline in learning and memory abilities and the formation of cognitive impairments.

The impact of alcohol on cognitive function can be explained through several key biological mechanisms. Firstly, alcohol affects cognitive function by disrupting neurotransmitter systems, especially by altering the balance between glutamate and gamma-aminobutyric acid (GABA) in the central nervous system (Rodriguez et al., 2022; Goodwani et al., 2017). Glutamate is the primary excitatory neurotransmitter, while GABA is the primary inhibitory neurotransmitter (Rodriguez et al., 2022; Goodwani et al., 2017). Alcohol modifies the activity of these neurotransmitters, significantly affecting neural transmission and brain function (Rodriguez et al., 2022; Goodwani et al., 2017). Secondly, the metabolic byproduct of alcohol, acetaldehyde, has cytotoxic properties that can directly damage brain cells, prompting an increase in inflammatory responses and oxidative stress (Makiko, 2020; Visontay et al., 2021; Ueno et al., 2022). These reactions not only damage neurons but also activate microglial cells, further increasing the release of inflammatory mediators, leading to neuroinflammation and brain tissue damage (Makiko, 2020; Visontay et al., 2021; Ueno et al., 2022). Furthermore, acetaldehyde also promotes the deposition of beta-amyloid, the aggregation of which is a key pathological process in Alzheimer’s disease and other types of cognitive disorders (Venkataraman et al., 2017; Veitch et al., 2022). The identification of these mechanisms underscores the importance of controlling alcohol intake, especially in populations vulnerable to alcohol’s effects, those at risk for cognitive impairments, and those with a family history of Alzheimer’s disease. By providing targeted interventions, such as cognitive-behavioral therapy and lifestyle adjustment guidance, it is possible to help reduce alcohol consumption and mitigate its negative impacts on cognitive function (Visontay et al., 2021; Kivipelto et al., 2018).

Additionally, this study clearly revealed a nonlinear relationship between alcohol intake and cognitive function through smooth curve fitting and threshold effect analysis, indicating that alcohol intake has a significant negative impact on cognitive function beyond a specific threshold. Particularly in the CERAD total word recall and Animal Fluency Tests, we identified specific alcohol intake inflection points, which mark the transition from potential protective effects of alcohol to its harmful impacts. Meanwhile, the findings of Zhang et al. support our results, noting a significant positive correlation between low to moderate drinking and slower cognitive decline and higher maintenance of cognitive function (Zhang et al., 2020). However, the findings of Topiwala et al. (2017) contrast with our observations of a nonlinear relationship, as they observed that even moderate alcohol intake is associated with hippocampal atrophy and continuous decline in cognitive function. Topiwala’s et al. (2017) results particularly emphasize that even universally recognized moderate levels of drinking can have long-term negative effects on neural structures, especially in individuals who are susceptible to the effects of alcohol.

The nonlinear relationship between alcohol intake and cognitive function suggests that alcohol may have protective effects within certain limits, but exceeding a specific threshold can lead to significant cognitive impairment. Moderate alcohol consumption may protect brain structure and function, and maintain cognitive health by enhancing vascular health, reducing inflammation, and regulating key neurotransmitter levels (Zhang et al., 2020; Serio et al., 2023; Hrelia et al., 2022). However, excessive alcohol demonstrates its neurotoxicity by promoting the production and deposition of beta-amyloid, as well as damaging neuronal synaptic structures, leading to cognitive impairment (Venkataraman et al., 2017; Veitch et al., 2022). Simultaneously, the findings of Topiwala et al. (2017) provide an important additional perspective on this complex relationship. Their study observed that even universally recognized moderate alcohol intake is associated with a reduction in the volume of specific brain areas, particularly the hippocampus, which is crucial for memory and cognitive functions (Topiwala et al., 2017). This hippocampal atrophy is related to continuous cognitive decline, indicating that even moderate alcohol consumption can have long-term negative effects on individual neural structure and function, especially in individuals who are sensitive or susceptible to alcohol’s effects (Dawe et al., 2020; Hanseeuw et al., 2023). In summary, the impact of alcohol on cognitive function is multi-layered, covering a wide range from potential protection to significant damage. These findings underscore the need for greater caution in recommendations regarding alcohol intake. For the elderly population and individuals at high risk for neurodegenerative diseases, even moderate alcohol intake should be strictly monitored to prevent its potential adverse effects. Healthcare providers need to consider the complex impact of individual alcohol consumption habits on cognitive health, especially when formulating public health policies and personalized health plans.

Finally, the subgroup analysis of this study also revealed significant moderating effects of gender, education level, and smoking status on the relationship between alcohol intake and cognitive function. Gender differences suggest that compared to women, men’s cognitive functions are more susceptible to the negative impacts of alcohol. This difference may be due to gender-specific biological metabolism and hormonal regulation, as well as cultural and societal structures that shape gender expectations regarding drinking behavior (Hanseeuw et al., 2023; Finn, 2023; Agardh et al., 2021; Kinjo et al., 2023; Brennan et al., 2020). For instance, women might be more sensitive to alcohol due to slower metabolic rates, yet moderate drinking in women may be associated with certain cognitive protective effects, potentially related to the protective roles of sex hormones like estrogen (Hanseeuw et al., 2023; Finn, 2023). The impact of education level reveals an intriguing phenomenon. Higher education levels do not seem to provide the expected protective effect; instead, they show that alcohol intake has a more significant negative impact on cognitive function in this group. This could be related to higher cognitive demands faced by individuals with higher education in their work and daily lives, making any potential cognitive impairment more noticeable and assessable (Brennan et al., 2020; Pachito et al., 2021). Conversely, individuals with lower education levels might be more sensitive to the slight positive effects of alcohol in the short term due to lower baseline cognitive demands (Brennan et al., 2020; Pachito et al., 2021). The interactions shown by smoking status are complex and thought-provoking. Non-smokers exhibited more pronounced negative effects of alcohol intake on cognitive function, while smokers showed some positive effects of alcohol on cognition to a certain extent (Hagger-Johnson et al., 2013). This may suggest a potential synergistic interaction between smoking and drinking, which could jointly affect cognitive functions through altering neuro biochemical responses or impacting cerebrovascular health (Hagger-Johnson et al., 2013). The exact mechanisms behind this phenomenon still require further investigation for clarification. Overall, these findings emphasize the necessity of formulating targeted public health policies that consider the impacts of factors such as gender, education, and lifestyle habits.

### 4.1 Strengths and limitations

This study utilized data from NHANES, conducted by (NCHS), which are highly representative and reliable, effectively reflecting the health status of the elderly population in the United States. Additionally, by employing multivariate regression analysis, smooth curve fitting, and threshold effect analysis, this study not only explored the linear relationship between alcohol intake and cognitive function but also revealed potential nonlinear relationships, adding depth and complexity to the analysis. Finally, through subgroup analysis of different factors such as gender, education level, and smoking status, this study provides more detailed insights, helping to understand the variations in the impact of alcohol intake on cognitive function across different populations.

This study also faces several limitations. First, due to the cross-sectional design of the NHANES survey employed, causal relationships cannot be directly inferred. Second, although this research has considered many potential confounders, there may still be unobserved variables such as socioeconomic status and other lifestyle factors that could affect the association between alcohol and cognitive function. Third, the alcohol consumption data in this study are based on self-reported intake by participants, which may be subject to recall bias, thus affecting the accuracy of the results. Unlike studies such as those by Fitzpatrick Schmidt et al., which precisely measure alcohol intake through Phosphatidylethanol (PEth) (Fitzpatrick-Schmidt et al., 2024), this study lacks such precise biomarker data, potentially limiting the accuracy of alcohol intake assessments. Fourth, in the process of categorizing participants as either heavy or non-heavy drinkers, although this classification facilitates data analysis, it might obscure subtle differences in drinking behaviors, thereby affecting the interpretation and generalizability of the results. This is particularly true within the non-heavy drinkers group, where certain drinking patterns may still exist. Finally, the study’s observations are limited to the elderly population in the United States, thus the findings may not be applicable to other ethnicities or populations.

Future research could explore the direct causal relationships between alcohol intake and changes in cognitive function through long-term prospective studies, while considering the impacts of long-term lifestyle changes and other relevant health factors. Second, based on the current findings, intervention studies could be designed to assess the potential benefits of reducing alcohol intake on the improvement or maintenance of cognitive functions. Third, future studies could overcome the limitations of self-reported data by using more precise tools such as biomarkers to measure alcohol intake. Fourth, it is recommended that future research adopt more detailed categorizations of alcohol intake to more accurately assess the relationship between alcohol consumption and cognitive function and to reveal more complex association patterns. Finally, employing advanced statistical techniques like machine learning to process large datasets may reveal more complex relationships and interaction patterns, thereby providing deeper insights into how alcohol affects cognitive function.

## 5 Conclusion

This study demonstrates that there is a nonlinear relationship between alcohol intake and cognitive function in the elderly, where moderate drinking may have a protective effect, but exceeding a certain threshold will have a negative impact on cognitive function. The significant moderating effects of gender, education level, and smoking status highlight the importance of considering individual differences when formulating public health policies. These findings support the moderate restriction of alcohol consumption and emphasize the need for public health to develop more refined health guidelines tailored to the specific circumstances of different populations.

## Data Availability

The original contributions presented in the study are included in the article/supplementary material, further inquiries can be directed to the corresponding author.

## Footnotes

^1^https://www.cdc.gov/nchs/nhanes/AnalyticGuidelines
